# Fitness, fatness and the reallocation of time between children’s daily movement behaviours: an analysis of compositional data

**DOI:** 10.1186/s12966-017-0521-z

**Published:** 2017-05-10

**Authors:** Stuart J. Fairclough, Dorothea Dumuid, Sarah Taylor, Whitney Curry, Bronagh McGrane, Gareth Stratton, Carol Maher, Timothy Olds

**Affiliations:** 1 Physical Activity and Health Research Group, Department of Sport and Physical Activity, Edge Hill University, St Helens Road, Ormskirk, Lancashire UK; 2 Department of Physical Education and Sports Science, University of Limerick, Limerick, Ireland; 3 Alliance for Research in Exercise Nutrition and Activity (ARENA), Sansom Institute, School of Health Sciences, University of South Australia, Adelaide, Australia; 4 School of Arts Education and Movement, Dublin City University, Institute of Education, St Patrick’s Campus, Dublin, Ireland; 5 Research Centre in Applied Sports, Technology Exercise and Medicine, College of Engineering, Swansea University, Swansea, Wales UK

**Keywords:** Sedentary time, Physical activity, Accelerometer, LPA, MVPA, Sleep

## Abstract

**Background:**

Movement behaviours performed over a finite period such as a 24 h day are compositional data. Compositional data exist in a constrained simplex geometry that is incongruent with traditional multivariate analytical techniques. However, the expression of compositional data as log-ratio co-ordinate systems transfers them to the unconstrained real space, where standard multivariate statistics can be used. This study aimed to use a compositional data analysis approach to examine the adiposity and cardiorespiratory fitness predictions of time reallocations between children’s daily movement behaviours.

**Methods:**

This study used cross-sectional data from the Active Schools: Skelmersdale study, which involved Year 5 children from a low-income community in northwest England (*n* = 169). Measures included accelerometer-derived 24 h activity (sedentary time [ST], light physical activity [LPA], moderate-to-vigorous physical activity [MVPA], and sleep), cardiorespiratory fitness determined by the 20 m shuttle run test, objectively measured height, weight and waist circumference (from which zBMI and percent waist circumference-to-height ratio (%WHtR) were derived) and sociodemographic covariates. Log-ratio multiple linear regression models were used to predict adiposity and fitness for the mean movement behaviour composition, and for new compositions where fixed durations of time had been reallocated from one behaviour to another, while the remaining behaviours were unchanged. Predictions were also made for reallocations of fixed durations of time using the mean composition of three different weight status categories (underweight, normal-weight, and overweight/obese) as the starting point.

**Results:**

Replacing MVPA with any other movement behaviour around the mean movement composition predicted higher adiposity and lower CRF. The log-ratio model predictions were asymmetrical: when time was reallocated to MVPA from sleep, ST, or LPA, the estimated detriments to fitness and adiposity were larger in magnitude than the estimated benefits of time reallocation from MVPA to sleep, ST or LPA. The greatest differences in fitness and fatness for reallocation of fixed duration of MVPA were predicted at the mean composition of overweight/obese children.

**Conclusions:**

Findings reinforce the key role of MVPA for children’s health. Reallocating time from ST and LPA to MVPA in children is advocated in school, home, and community settings.

**Electronic supplementary material:**

The online version of this article (doi:10.1186/s12966-017-0521-z) contains supplementary material, which is available to authorized users.

## Background

Evidence suggests that childhood physical activity and sedentary behaviour are independently associated with a range of health indicators [1, 2]. Paediatric physical activity research has traditionally focused on the health benefits of moderate-to-vigorous physical activity (MVPA) but in recent years there has been increased attention given to light physical activity (LPA) [3–6]. LPA represents the majority of physical activity accumulated by children during waking hours and some studies have found associations with adiposity [6], fitness [4], and cardiometabolic health [3, 5]. Similarly, increased interest is now paid to the influence of sedentary behaviours on health in youth [2, 7–9]. Physical activity subcomponents and sedentary behaviour are specific foci of activity guidelines in the UK [10], Canada [11], Australia [12], and elsewhere. However, a recent shift has seen sleep included in the 2016 Canadian 24 h movement guidelines for children and youth which integrate physical activity, sedentary behaviour, and sleep [13]. In children, sleep is associated with adiposity, emotional regulation, academic achievement, and quality of life, and is seen as critical to health and wellbeing [14]. The Canadian guidelines were the first to provide evidence-informed recommendations for a healthy 24 h day which includes MVPA, LPA, sedentary behaviours, and sleep [13].

The premise for the 24 h movement guidelines concept is that certain combinations of movement behaviours rather than attainment of a single recommendation, such as 60 min of daily MVPA, may contribute more fully to child health [15, 16]. This approach is consistent with the notion that to properly understand the relationships between health and movement behaviours, the effects of physical activity, sedentary behaviour, and sleep should be studied relative to each other rather than in isolation [17, 18]. Recently, a small number of studies have applied this concept by treating movement behaviour data as compositional data [19, 20]. Compositional data are made up of mutually exclusive and exhaustive parts of a whole [17]. Movement behaviours (sleep, sedentary time (ST), LPA, and MVPA) collectively constitute the entire 24 h day. Accordingly, movement behaviours exist in a sample space restricted by a constant sum constraint of 24 h [20]. The constant sum constraint imposes perfect multi-collinearity among behaviours; i.e., to maintain the constant total sum of 24 h, one movement behaviour cannot change without a corresponding change in one or more of the remaining behaviours. The sample space occupied by compositional data is termed the *Simplex* and is subject to a specific geometry (*Aitchison* geometry) which is incongruent with conventional multivariate statistical techniques designed to operate in unconstrained, *Real* space governed by *Euclidean* geometry [17]. However, using the principles of compositional data analysis proposed by Aitchison [17] and pioneered in activity research by Chastin et al. [20], compositional data can be transferred to the unconstrained *Real* Space using a log-ratio transformation. A number of transformations have been described, however only the isometric log-ratio transformation ensures that the relative difference between components is maintained, and that the resulting isometric log-ratio co-ordinates are orthogonal (i.e., not multi-collinear) [20]. Once compositional data are expressed as isometric log-ratio coordinates, conventional statistical methods (e.g., multiple linear regression) can be applied. Thus, compositional data analysis accounts for the collinear and co-dependent interactions between movement behaviours performed over a finite time period such as a 24 h day [17], and when examining associations with health outcomes allows for correct adjustment of all behaviours on the movement continuum [20]. Compositional data analysis therefore offers researchers investigating relationships between physical activity, sedentary behaviour, sleep, and health an improved means of dealing with the intrinsically compositional nature of movement behaviour data. Recently, Carson et al. used compositional data analysis to investigate the health associations between self-reported sleep, objectively measured activity behaviours and a range of health indicators among a large sample of Canadian children and youth [19]. The study reinforced the importance of MVPA for optimal health in children and youth, and also provided support for the importance of time spent in movement behaviours other than MVPA [19]. The aim of the present study was to use compositional data analysis to examine the adiposity and cardiorespiratory predictions when reallocating fixed durations of time between movement behaviours in a sample of English children. The study is one of only a small number to apply a compositional data analysis approach novel to accelerometer-measured estimates of children’s daily physical activity, sedentary behaviour, and sleep.

## Methods

### Participants

Two hundred and fifteen children aged 9–10 years from seven primary schools participated in the baseline phase of the cross-sectional Active Schools: Skelmersdale (AS:Sk) study. The schools were situated in Skelmersdale, which is a low income town within the West Lancashire region of northwest England [21]. All 15 primary schools in the town were invited to participate in the project. Twelve schools initially expressed interest and seven schools committed to take part. These schools were situated in districts where average overweight and obesity prevalence in 10–11 year olds exceeded 33% [21]. All Year 5 children (*n* = 243) were invited to take part in the study and 215 returned signed parent/carer informed consent and child assent (75% participation rate). The study received ethical approval from Edge Hill University’s Faculty of Arts and Sciences Research Ethics Committee.

### Measures

#### Anthropometry

Stature was assessed to the nearest 0.1 cm using a portable stadiometer (Leicester Height Measure, Seca, Birmingham, UK). Body mass was assessed to the nearest 0.1 kg (761 scales, Seca, Birmingham, UK). Body mass index (BMI) was calculated for each child, with BMI z-scores (zBMI) also assigned [22]. Age- and sex-specific BMI cut points classified children as underweight, normal-weight, or overweight/obese [23]. Waist circumference was measured to the nearest 0.1 cm using an anthropometric tape measure that was extended around the waist while the children were breathing normally, and the percentage of waist circumference-to-height ratio (%WHtR) was calculated as a measure of central adiposity [24]. All measurements were taken by the third author and a research assistant using standard procedures.

#### Socio-economic status

Neighbourhood-level socio-economic status (SES) was calculated using the UK Government 2015 Indices of Multiple Deprivation (IMD) [25]. IMD rank scores were generated using the National Statistics Postcode Directory database from parent reported home postcodes. IMD rank scores were matched to their corresponding IMD deciles, where decile 1 represents the most deprived 10% of areas nationally.

#### Cardiorespiratory fitness

The 20 m shuttle run test was conducted to provide an estimate of cardiorespiratory fitness (CRF) [26]. This test has been widely used in children of similar age [27, 28]. The running speed at the last completed lap was used to estimate peak oxygen uptake (VO_2_ peak; ml·kg·min^−1^) using the Léger et al. prediction equation [28].

#### Activity composition

Free-living activity composition was assessed using the ActiGraph GT9X triaxial accelerometer (ActiGraph, Pensacola, FL) worn on the non-dominant wrist for 24 h·day^−1^ for 7 days, except for water-based activities. The GT9X monitor uses the same MEMS sensor as the GT3X+ model which has been used extensively with children. Log sheets were provided to record times when the accelerometers were removed and replaced. Data collection took place during the regular school term from May to July 2016 so data were representative of usual spring/summer free-living activities. Data, sampled at 30 Hz, were analysed using ActiLife v. 6.11.5 (ActiGraph, Pensacola, FL) and saved in raw format as GT3X files. The raw data files were processed in R (http://cran.r-project.org) using the GGIR package (version 1.2–11) which autocalibrated the raw triaxial accelerometer signals [29]. Signals were then converted into gravity-corrected vector magnitude units, termed the Euclidean norm minus one (ENMO) [30], which were expressed as the average ENMO values per 1 s epoch.

Accelerometer wear time inclusion criteria were a minimum of 16 h∙day^−1^ for at least any 3 days. Non-wear was estimated on the basis of the standard deviation and value range of each accelerometer axis, calculated for moving windows of 60 min with 15 min increments [30], which has been applied previously in ActiGraph studies involving children [31–33]. For each 15 min period detected as non-wear time over the valid days, missing data were replaced by the mean value calculated from measurement on other days at the same time of day [34, 35]. Application of the wear time inclusion criteria resulted in an analytical sample of 169 children (78.6% of consenting children), whose descriptive characteristics did not differ from those of the excluded children. Hildebrand et al.’s prediction equations were used to identify cut-points for classifying activity into ST, LPA, and MVPA [36]. In youth 2 METs [37] and 4 METs [38] thresholds have better classification accuracy for differentiating ST (from LPA) and MVPA (from LPA), respectively, compared with adult values of 1.5 METs and 3 METs. Therefore, the Hildebrand equations were solved for 2 METs (ST/LPA) and 4 METs (MVPA). Sleep was estimated from the ActiGraph raw accelerations and arm angle using the method of van Hees and colleagues [39] within the GGIR R package (version 1.2–11; http://cran.r-project.org).

### Data analysis

Analyses were performed in R (http://cran.r-project.org) using the compositions package (version 1.40–1) [40]. Compositional data (average daily time spent in sleep, ST, LPA and MVPA) were expressed as isometric log-ratio co-ordinates using the default isometric log-ratio transformation, ilr(), included in the compositions package. There are many possible isometric log-ratio transformations [41], however for the purpose of the ensuing analysis, the type of isometric log-ratio is inconsequential, i.e., the results will be the same regardless of the partitioning system used to create the isometric log-ratio. However, it is important to ensure the same partitioning system is used when an *inverse* isometric log-ratio transformation is applied to transfer data back to the Simplex space.

Once the compositional movement behaviours were expressed as log-ratio co-ordinates, conventional descriptive statistics were applied. Arithmetic means of the isometric log-ratio co-ordinates were computed in Real space. The means were then back-transferred to the Simplex (i.e., expressed as movement behaviours) via the default inverse isometric log-ratio function, ilrInv(). Subsequently, the means were adjusted to collectively sum to 1440 min (24-h) to describe the central tendency of the 24 h movement behaviours. Central tendency of 24-h compositional data can equally be determined by calculating the geometric mean of each component and adjusting the resulting means to sum to 1440 min, as described by Chastin et al. [20]. The multi-variate dispersion of the daily movement behaviours was described by a pair-wise variation matrix [17]. Conventional univariate measures of dispersion such as standard deviations are unable to capture the co-dependent nature of compositional data [17]. The variation matrix was derived by calculating the variation of the logarithms of all possible pair-wise ratios (e.g., variation of *ln*(Sleep/MVPA)). A smaller variation element indicates more consistent proportionality between the two activities.

Multiple linear regression models were used to investigate the adiposity and fitness associations of time reallocation from one movement behaviour to another. Movement behaviours were expressed as isometric log-ratio co-ordinates (using the default ilr() transformation) and used as explanatory variables in the linear models. Sociodemographic covariates (sex, age, and IMD decile) were also included as explanatory variables. The outcome variables were zBMI, (2), %WHtR and (3) VO_2_ peak. Subsequent VO_2_ peak analyses were performed with zBMI as an additional covariate to explore the contribution of composition-related fatness to differences in CRF. The isometric log-ratio multiple linear regression models were checked for linearity, normality, homoscedasticity and outlying observations to ensure assumptions were not violated. The significance of the explanatory variables was examined with the car::Anova() function, which uses Wald Chi squared to calculate Type II tests according to the principle of marginality, testing each covariate after all others [42].

The linear models were used to predict adiposity (zBMI, %WHtR) and CRF (VO_2_ peak) for a baseline composition (i.e., the mean daily movement behaviour composition of all children), expressed as isometric log-ratio co-ordinates. Subsequently, predictions were calculated for new compositions (also expressed as isometric log-ratio co-ordinates), where fixed durations of time had been reallocated from one movement behaviour to another while the remaining behaviours were kept constant. Predictions were calculated for a range of compositions, with reallocations of time from 0 to 25 min, in 5 min increments. A simple subtraction calculation was used to find the difference in fatness and fitness predicted for the mean composition and the new compositions. For example, consider the mean composition C_mean_ = (sleep_mean_, ST_mean_, LPA_mean_, MVPA_mean_), expressed as isometric log ratio coordinates, with a predicted zBMI of zBMI_mean_. Now, consider a new composition C_10_, where 10 min has been reallocated from sleep to MVPA, i.e., C_10_ = (sleep_mean – 10 min_, ST_mean_, LPA_mean_, MVPA_mean + 10 min_), also expressed as isometric log-ratio co-ordinates, with a predicted zBMI of zBMI_mean10_. The predicted difference in zBMI for the 10 min reallocation is derived by subtracting zBMI_mean_ from zBMI_mean10_. This procedure was repeated for all possible pair-wise combinations of activity behaviours. A detailed description of this analysis and example R code is included in Additional file 1. This procedure differs from the change-matrix methodology pioneered by Chastin et al. [20], where the linear model regression coefficients for the isometric log-ratio co-ordinates are first transferred to the Simplex space via the inverse isometric log-ratio transformation.

As each new composition is constructed to maintain the constant sum of 1440 min (or 24 h), the reallocation of a fixed duration of time from one behaviour to another behaviour can mathematically be conceptualised as a form of *compositional isotemporal substitution*, distinct from *traditional* isotemporal substitution [43], which treats compositional data in an incompatible Euclidean geometry. However, it must be acknowledged that due to the cross-sectional nature of the present study, predictions from the linear models do not represent the change in fitness and fatness with isotemporal changes in an individual’s daily composition. Rather, fitness and fatness are predicted for individuals with specific daily movement behaviour compositions where fixed durations of time have been reallocated between behaviours. The results of compositional isotemporal substitution (unlike traditional isotemporal substitution) will differ according to the baseline activity composition. Predicted differences were therefore calculated for the mean activity behaviour compositions of (1) underweight, (2) normal-weight, and (3) overweight/obese children.

## Results

The mean age of the children was 10.3 y and around half were girls (Table 1). Sixty-eight percent of the children were categorized as normal-weight and the proportion of overweight/obese was 23%. Eighty-five percent of the children lived in areas of high relative deprivation (IMD deciles 1–3). On average the children wore the accelerometers for at least 16 h for 5.9 days, and the mean accelerometer wear time was 23.4 h∙day^−1^.

**Table 1:** Participant characteristics

	All (*n* = 169)	Underweight (*n* = 15)	Normal-weight (*n* = 115)	Overweight/obese (*n* = 39)
Age (y)	10.3 (.3)	10.1 (.3)	10.3 (.03)	10.3 (.3)
Sex (%)				
Boys	49.7	53.3	51.3	43.6
Girls	50.3	46.7	48.7	56.4
Height (cm)	141.2 (6.2)	136.5 (3.9)	140.3 (5.4)	145.5 (7.1)
Weight (kg)	37.3 (9.8)	26.3 (2.1)	33.9 (4.2)	51.5 5 (8.9)
BMI (kg·m^2^)	18.5 (3.7)	14.1 (.8)	17.2 (1.5)	24.2 (2.7)
zBMI	0.43 (1.31)	−1.74 (.89)	0.10 (.71)	2.26 (.50)
Waist-circumference (cm)	64.3 (10.3)	55.4 (2.6)	60.8 (6.8)	78.2 (7.6)
%WHtR	46 (6)	41 (2)	44 (3)	54 (5)
CRF (laps)	28.8 (14.7)	36.3 (16.9)	32.1 (13.8)	16.2 (8.1)
VO_2_ peak (ml·kg·min^−1^)	47.0 (4.0)	49.6 (4.0)	47.9 (3.7)	43.3 (2.6)
IMD decile	2.5 (2.0)	2.8 (2.7)	2.4 (1.9)	2.6 (1.9)

Data are presented as mean ± SD for continuous variables and percentage for sex as a categorical variable

*BMI* body mass index; *zBMI* body mass index z-score; *%WHtR* percentage waist circumference-to-height ratio; *CRF* cardiorespiratory fitness; *VO*_2_ *peak* peak oxygen uptake; *IMD* indices of multiple deprivation

Compositional means for activity behaviours for the full sample and for the weight status groups are shown in Table 2. In the full sample the compositional means indicated that 38% of the 24-h period was spent in sleep, 35% in sedentary time, 25% in LPA, and 2% in MVPA. ST was higher, and LPA and MVPA were lower in the overweight/obese group relative to the underweight and normal-weight children.

**Table 2:** Geometric means for sleep, ST, LPA, and MVPA

	All (*n* = 169)	Underweight (*n* = 15)	Normal-weight (*n* = 115)	Overweight/obese (*n* = 39)
Sleep (min·day)^−1^	548.6	546.0	545.6	557.1
ST (min·day)^−1^	510.3	488.1	509.3	520.9
LPA (min·day)^−1^	354.8	370.9	356.2	343.9
MVPA (min·day)^−1^	26.4	35.0	28.9	18.1

Activity behaviours are presented as compositional means, adjusted to sum to 1440 min·day^−1^

*ST* Sedentary Time; *LPA* Light Physical Activity; *MVPA* Moderate-to-Vigorous Physical Activity

The variability of the data for the full sample is summarized in the compositional variation matrix (Table 3). Values close to zero indicated that the times spent in the two behaviours included in the ratio were highly co-dependent. The smallest variances were observed for sleep and ST, for sleep and LPA, and for ST and LPA. These values of <0.08 imply high co-dependence between each pair of variables. The highest variances were observed for MVPA, which demonstrated that time spent in MVPA was the least co-dependent on the other behaviours. Compositional variation matrices for each weight status group can be found in Additional file 2.

**Table 3:** Compositional variation matrix of time spent by the full sample in sleep, ST, LPA, and MVPA

	Sleep	ST	LPA	MVPA
Sleep	0	0.030	0.036	0.281
ST	0.030	0	0.079	0.442
LPA	0.036	0.079	0	0.235
MVPA	0.281	0.442	0.235	0

*ST* Sedentary Time; *LPA* Light Physical Activity; *MVPA* Moderate-to-Vigorous Physical Activity

Analysis of variance of multiple linear regression model parameters indicated that the isometric log-ratio co-ordinates were statistically significant predictors of zBMI, %WHtR and CRF (Table 4). Table 5 displays the predicted difference in zBMI, %WHtR, and CRF when 15 min was reallocated from the behaviours in the columns to the behaviours in the rows, keeping the remaining behaviours constant. For example, in the full sample, children with 15 min less MVPA and 15 min more ST had a predicted zBMI which was 0.83 units higher than the predicted mean zBMI (see Additional file 3 for predicted mean zBMI, %WHtR, and VO_2_ peak values). Of the 15 min reallocations, the largest differences in predicted fatness and CRF were observed for children with lower MVPA, in favour of any other behaviour. The opposite reallocations (higher MVPA and lower sleep, ST and LPA) predicted the opposite fatness and CRF associations (lower zBMI, lower WHtR and higher CRF), however these relationships were asymmetrical. For example, predicted zBMI was reduced by a smaller amount with the addition of 15 min *extra* MVPA (−0.43 to −0.49) than the increase in zBMI predicted for 15 min *less* MVPA (+0.83 to +0.89). Predicted changes in zBMI, %WHtR, and VO_2_ peak for increasing reallocations (from 2.5 min to 25 min) are presented in Additional files 4, 5 and 6, respectively. The pattern of predictions was similar when reallocations were performed using the daily movement composition of normal-weight children as a starting point. The magnitude of predictions were greatest when the daily movement composition of overweight/obese children was used as a starting point for reallocations. At their mean composition, reallocating 15 min from MVPA to sleep, ST, and LPA was associated with predicted increases in zBMI of 1.82, 1.77, and 1.83 units, respectively.

**Table 4:** Linear models for adiposity and cardiorespiratory fitness: Analysis of Variance

	Sum Sq	df	F value	Pr(>F)
zBMI				
Isometric log-ratio co-ordinates	32.0	3	7.0	<0.001
IMD decile	9.9	7	0.9	0.49
Sex	3.5	1	2.3	0.13
Age	6.9	1	4.6	0.03
%WHtR				
Isometric log-ratio co-ordinates	1123.2	3	9.9	<0.001
IMD decile	276.5	7	1.0	0.40
Sex	150.6	1	4.0	0.05
Age	45.0	1	1.2	0.28
VO_2_ peak				
Isometric log-ratio co-ordinates	708.0	3	20.6	<0.001
IMD decile	201.7	7	2.5	0.02
Sex	3.9	1	0.3	0.56
Age	4.4	1	0.4	0.54
VO_2_ peak with zBMI				
Isometric log-ratio co-ordinates	408.4	3	14.0	<0.001
zBMI	278.9	1	28.6	<0.001
IMD decile	184.8	7	2.7	0.01
Sex	0.0	1	0.0	0.99
Age	0.6	1	0.1	0.81

**Table 5:** Predicted differences in zBMI, %WHtR, and CRF following reallocation of 15 min between movement behaviours

	zBMI				%WHtR				CRF (VO_2_ peak, ml·kg·min^−1^) ^a^			
	Sleep	ST	LPA	MVPA	Sleep	ST	LPA	MVPA	Sleep	ST	LPA	MVPA
All children *n* = 169												
Sleep		0.05	−0.01	0.88		0.1	−0.4	5.1		0.0	−0.0	-2.4
ST	-0.05		−0.06	0.83	−0.1		−0.5	5.0	0.0		−0.0	-2.4
LPA	0.01	0.06		0.89	0.3	0.4		5.5	0.0	0.0		-2.4
MVPA	-0.48	−0.43	−0.49		−2.8	−2.7	−3.1		1.3	1.3	1.3	
Underweight children *n* = 15 ^b^												
Sleep		0.05	−0.01	0.59		0.1	−0.3	3.4		0.0	−0.0	-1.6
ST	-0.05		−0.06	0.54	−0.1		−0.4	3.3	0.0		−0.0	-1.6
LPA	0.01	0.06		0.60	0.3	0.4		3.7	0.0	0.0		-1.6
MVPA	-0.39	−0.33	−0.40		−2.2	−2.1	−2.5		1.1	1.1	1.0	
Normal weight children *n* = 115												
Sleep		0.05	−0.01	0.77		0.1	−0.4	4.5		0.0	−0.0	-2.1
ST	-0.05		−0.06	0.72	−0.1		−0.5	4.4	0.0		−0.0	-2.1
LPA	0.01	0.06		0.78	0.3	0.4		4.8	0.0	0.0		-2.1
MVPA	-0.45	−0.40	−0.46		−2.6	−2.5	−2.9		1.2	1.2	1.2	
Overweight/obese children *n* = 39												
Sleep		0.05	−0.01	1.82		0.1	−0.4	10.8		0.0	−0.0	-5.1
ST	-0.05		−0.06	1.77	−0.1		−0.5	10.7	0.0		−0.0	-5.1
LPA	0.01	0.06		1.83	0.4	0.5		11.1	0.0	0.0		-5.0
MVPA	-0.64	−0.59	−0.65		−3.7	−3.6	−4.1		1.8	1.8	1.7	

Estimation of change in zBMI, %WHtR or VO_2_ peak when the behaviour in the rows substitutes the behaviour in the columns

*zBMI* Body Mass Index z-score; *CRF* cardiorespiratory fitness; *VO*_2_ *peak* peak oxygen uptake; *ST* Sedentary Time; *LPA* Light Physical Activity; *MVPA* Moderate-to-Vigorous Physical Activity

Analysis adjusted for IMD decile, age, and sex. ^a^ Analysis adjusted for IMD decile, age, sex, and zBMI. ^b^ Underweight results reported for presentation purposes only due to low sample size

**Fig. 1:**
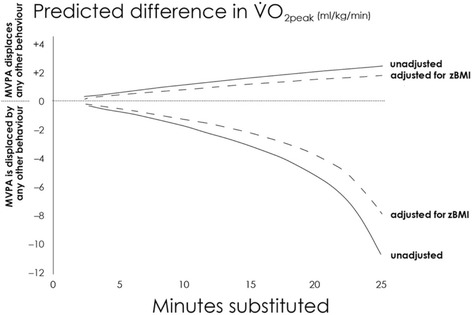
Difference in predicted CRF (VO_2_ peak) with reallocation of MVPA. Note. All analyses adjusted for IMD decile, age, sex, and zBMI

**Fig. 2:**
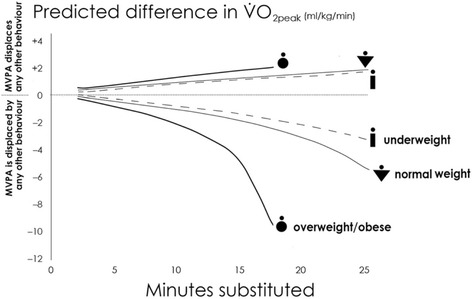
Difference in predicted CRF (VO_2_ peak) with reallocation of MVPA at various baseline compositions. Note. All analyses adjusted for IMD decile, age, sex, and zBMI

Figure 1 shows predicted CRF with time reallocations between behaviours with additional adjustment for zBMI to fractionate out the effect of body mass on CRF. It was found that adjusting for zBMI attenuates the differences in predicted CRF with time reallocations involving MVPA. However, a major residual association between reallocations and predicted CRF was observed. Reallocations of time around the mean movement behaviour compositions of the three weight status groups revealed that differences in predicted CRF were largest when reallocations were performed using the mean composition for overweight/obese children as a starting point (Fig. 2). The non-linearity of predicted fatness and CRF over increasing time reallocations was particularly evident when MVPA was displaced by other behaviours. Unfavourable predictions escalated when the amount of time reallocated away from MVPA exceeded 15 min at the normal-weight composition, however at the overweight/obese composition, escalation in unfavourable predictions was seen at approximately 10 min reallocations from MVPA.

## Discussion

This study used the principles of compositional data analysis to predict adiposity and CRF for reallocations of time between daily movement behaviours in a sample of English children. The study adds to the emerging body of research acknowledging the compositional nature of 24 h device measured estimates of sleep, ST, LPA, and MVPA. The findings reinforce the importance of MVPA for primary school aged children’s CRF and adiposity. We found that replacing MVPA with any other movement behaviour around the average composition predicted higher adiposity and lower CRF. The magnitude of predicted differences was similar when time was reallocated from MVPA to either sleep, ST, or LPA. The largest predicted differences were for zBMI, whereby reallocating 15 min from MVPA to the other behaviours was associated with a substantial predicted change of +0.83–0.89 units from mean zBMI compared to smaller predicted changes in %WHtR (+5.0 to 5.5 percentage points), and CRF (−2.4 ml·kg·min^−1^). In the only other movement behaviour compositional isotemporal substitution analyses reported to date, for a 10 min substitution of MVPA for ST, zBMI and CRF changes of +5% and −0.05%, respectively were reported in children [19], and a + 1.2% change in BMI was reported in adults [20]. MVPA derived from wrist-worn accelerometers in our study was anchored to 4 METS and had a relatively low whole group compositional mean of 26.4 min, compared to 50.7 min as reported by Carson and colleagues who used a 3 MET hip-worn accelerometer MVPA threshold [19]. Consequently, a 15 min change in MVPA in our sample represented a change of over half (57%) of the compositional mean, which is much larger than the change for 10 min substitution reported in the Canadian sample [19]. The fact that compositional data analysis is referent to the mean composition explains why the magnitude of the associations we observed were substantially greater than those reported previously. Such comparisons are further complicated by use of different accelerometer data processing and wear protocols. Negligible changes in predicted zBMI, %WtHR, and CRF were seen in compositional isotemporal substitutions that did not involve MVPA. Similar findings have been reported for a range of cardiometabolic risk indicators in children [19] and in adults [20].

The relationships between reallocated sleep, ST, or LPA for MVPA around the average composition were asymmetrical, whereby the magnitudes of change in predicted adiposity and CRF were lower when MVPA replaced the other behaviours. Similar results were reported for compositional isotemporal substitution analyses in children [19] and adults [20], respectively. It is posited that the relative contributions of sleep, ST, LPA, and MVPA to the 24 h day partially explain the asymmetrical nature of the relationships. Taking time from MVPA which only contributes a small amount to a 24 h day (1.8% in our sample) is a substantially larger relative change than taking time away from ST for example, which contributes a larger segment of the 24 h day (35% in our sample) [19, 20]. Moreover, physiological adaptations associated with overloading exercise capacity (e.g., from doing more MVPA) occur slowly resulting in smaller health gains, compared to the more rapid effects of detraining and reversibility (e.g., from increasing ST) which are likely to negatively impact health in a more significant manner [19]. Our findings reinforce the notion that children should maintain or increase their level of MVPA engagement to avoid significant and inverse effects on adiposity and fitness status. This may be particularly important for the children in our sample who were transitioning from primary school to secondary school when declines in physical activity [44, 45] related to maturity status [46], physical self-perceptions [47], and in-school and after-school physical activity provision [48, 49] often occur.

Although the predicted favourable differences in adiposity and CRF for reallocation of time from sleep, ST, or LPA to MVPA were smaller than the unfavourable differences predicted for reallocations of time away from MVPA, the magnitudes of these predictions were still meaningful. For example, we observed changes in predicted zBMI and %WtHR of −0.43 and −2.65, respectively when ST was replaced with MVPA at the mean daily behaviour composition. These are greater than significant reductions in zBMI and WtHR values reported in intervention studies among obese [50–53] and non-obese children [54–56]. Similarly, when considering the positive change in predicted CRF as a result of reallocating sleep, ST, and LPA to MVPA, the 1.3 ml·kg·min^−1^ difference would be sufficient to shift a 10 y old child up into the next decile of recently published international normative VO_2_ peak values [28]. Adiposity and CRF are important predictors of future health status which track moderately from childhood through adolescence [57] and into adulthood [58, 59]. Furthermore, both are significantly associated with cardiometabolic risk with adiposity mediating the relationship between CRF and risk [60, 61]. Thus, when the effects on adiposity and CRF are considered together, the health implications of shifting children’s movement behaviours from inactive ones to MVPA are significant [16, 62]. However, in practice it would be inappropriate to advocate replacing sleep with MVPA because of the important role of sleep in children’s health and development [63].

We did not observe favourable predictions for adiposity or CRF when sleep and ST were replaced with LPA. This is consistent with Carson et al. who reported negligible effects on zBMI and CRF when 10 min of sleep or ST were reallocated to LPA [19]. Moreover, a recent non-compositional isotemporal substitution analysis saw a small increase in body fat when 60 min of ST was replaced with LPA in a sample of English children [64]. These findings also concur with a recent systematic review of objectively measured youth physical activity and health indicators, where LPA relationships with CRF and adiposity were mixed [65]. It is suggested that differences in accelerometer LPA cutpoints contribute to these inconsistent results [65] and that focusing on the upper end of the LPA intensity range may be more important when attempting to understand relationships between LPA and health indicators in youth [3].

The largest predicted differences in adiposity and CRF were seen when the mean composition for overweight/obese children was used as a baseline for reallocations of time between behaviours. When MVPA was reallocated to sleep, ST, or LPA around the mean overweight/obese composition, the differences in predicted zBMI, %WHtR, and CRF were more than double those observed for reallocations at the mean composition for normal-weight children. Overweight/obese children typically have lower CRF [66], motor competence [67, 68], physical self-perceptions [69, 70], and are less physically active than leaner peers [71, 72]. Our results demonstrate that from a health perspective this group have most to lose when their activity behaviours are replaced with less intense or sedentary ones. Moreover, when MVPA was displaced by other behaviours we observed a residual effect on CRF that was not attributable to adiposity. This suggests that children with various compositions were less fit not only because they were fatter, but because of other effects. As well as heredity, modifiable activity-related factors, such as vigorous physical activity [73], sports participation [74, 75], motor skill competence [76], active travel [74, 77], time spent outdoors [78], and screen time [77] are known to influence youth CRF. Providing intensity-appropriate and enjoyable physical activity opportunities for all children and for overweight/obese children in particular, is therefore important to positively impact CRF.

Our findings demonstrate the significant influence of MVPA on children’s adiposity and CRF. They also amplify the notion that optimal health may be achieved through combinations of MVPA alongside other movement behaviours which interact with one another [79]. For example, youth meeting guidelines for recommended durations of daily MVPA, sedentary time through recreational screen viewing, and sleep are likely to maximise their health benefits [16]. This approach underpins the 2016 Canadian 24 h movement guidelines and may represent a paradigm shift in the way that physical activity guidelines for health are operationalised, by considering the beneficial effects of activity beyond achieving the MVPA guideline. However, considering the small proportion of youth that engage in 60 min MVPA·day^−1^ [80], practitioners, policy-makers, health promotion workers, and researchers should also recognize the importance of providing opportunities that enable children to shift movement behaviours from ST and LPA to MVPA, and then maintain MVPA. The feasibility of enabling such reallocation of movement behaviours depends largely on the settings and contexts in which the behaviours occur. The school day provides a range of discretionary (e.g., active travel, recess play, after-school activities) and mandatory opportunities (e.g., physical education, classroom learning) for children to accrue MVPA. Comprehensive School Physical Activity Promotion programs are recognized as effective means of expanding, extending, and enhancing [81] these opportunities [82–85]. Out-of-school and weekend MVPA levels are typically lower than during school [86, 87] and are more strongly influenced by family and peers [88–90]. Promoting opportunities for MVPA in home and community settings that acknowledge the key roles of parents in particular, may complement more structured promotion efforts through schools.

A strength of this study is that children’s objectively measured 24 h movement behaviours were treated as compositional data. The appropriate analysis of compositional data adjusts for all collinear and co-dependent movement behaviours occurring over the finite 24 h period [18], while objective measurement of movement behaviours provides more accurate estimates than self-report methods [91]. A limitation though was that ST, LPA, and MVPA were based on acceleration thresholds that were not individually calibrated to the sample. Furthermore, the ST/LPA threshold was not informed by a measure of postural allocation, and sleep time was based on sustained inactivity bouts within a predefined angular range of arm motion [39] rather than using polysomnography. Thus, the resultant estimates of time spent in each movement behaviour could lead to different results compared to studies using alternative objective methods or thresholds. Although monitoring compliance was relatively high (79%) the homogenous nature of the sample suggest that the generalisability of the findings may be limited. Finally, due to the cross-sectional nature of the study, the predicted differences in fitness and fatness reflect more a sample shift in movement behaviour time allocations than actual differences for individuals [20].

## Conclusions

The novel compositional approach to the analysis of 24 h movement data allows the examination of co-dependent daily behaviours. Our findings add to the emerging body of research employing compositional data analysis to better understand the impact of different movement behaviours on indicators of health. We found that replacing MVPA with any other movement behaviour around the mean movement composition predicted higher adiposity and lower CRF. When MVPA substituted sleep, ST, or LPA, the predictions were asymmetrical. Compared to reallocations of time at the mean composition for normal-weight and underweight children, the magnitude of the predicted differences in fitness and fatness were greatest for reallocations at the overweight/obese children’s mean daily composition. The findings reinforce the key role of MVPA for children’s health and in particular, for overweight/obese children who are at greatest risk of chronic diseases later in life. Reallocating time from ST and LPA to MVPA in children is advocated in school, home, and community settings.

## Additional files

Additional file 1: Compositional data analysis and example R code. (DOCX 20 kb)

Additional file 2: Variation matrices of weight-status subgroups. (DOCX 13 kb)

Additional file 3: Predicted means for fatness and fitness used as the starting point for isotemporal substitutions. (DOCX 12 kb)

Additional file 4: Differences in predicted zBMI associated with time reallocations for the full sample. (DOCX 15 kb)

Additional file 5: Differences in predicted %WHtR associated with time reallocations for the full sample. (DOCX 14 kb)

Additional file 6: Differences in predicted VO_2_ peak associated with time reallocations for the full sample. (DOCX 14 kb)

Additional file 7: Study data. (XLSX 42 kb)

Additional file 8: STROBE checklist. (DOCX 30 kb)

## Abbreviations

CRF: Cardiorespiratory fitness
ENMO: Euclidean norm minus one
IMD: Indices of multiple deprivation
LPA: Light physical activity
MVPA: Moderate-to-vigorous- physical activity
ST: Sedentary time
WHtR: Waist-to-height-ratio
zBMI: Body mass index z-score
