# Supplementary material for: Fitness, fatness and the reallocation of time between children’s daily movement behaviours: an analysis of compositional data
Source: Int J Behav Nutr Phys Act. 2017 May 10;14:64. doi: 10.1186/s12966-017-0521-z (PMC5424384; doi:10.1186/s12966-017-0521-z)
Supplement: Supplementary file 1 — Compositional data analysis and example R code. (DOCX 20 kb) [file 12966_2017_521_MOESM1_ESM.docx]

**Additional file 1.**

*Using the isometric log-ratio regression model to calculate the difference in predicted outcomes when time is reallocated between movement behaviours.*

As outlined in the main text, the isometric log-ratio (*ilr)* linear multiple regression model can be used to predict health measures (e.g., zBMI) for daily movement behaviour compositions of interest. The *ilr* multiple linear regression used to model zBMI, *Y_i_*, for subjects *i=1,2,…n,* takes the form:

$$Y_{i}= \beta_{0}+\boldsymbol{\beta}^{T}ilr\boldsymbol{(}\boldsymbol{x}_{i}\boldsymbol{)} + \varepsilon_{i} (1)$$

where $\beta_{0}$is the intercept, $\boldsymbol{\beta}^{T}\boldsymbol{=}\left( \beta_{1},\beta_{2},\ldots,\beta_{D-1} \right)$ is a vector of coefficients, $\boldsymbol{x}_{i}\boldsymbol{=}\left( x_{i1},x_{i2},\ldots,x_{iD} \right)^{T}$ are the *D* observed components of the composition for subject *i*, $ilr\left( \boldsymbol{.} \right)$ is the isometric log-ratio function that translates the composition from the *D*-dimensional Simplex to the (*D*-1)-dimensional Euclidean space, and$\varepsilon_{i}$ is a zero-centred, independently normally distributed random variable with variance $\sigma^{2}$.

*Predicting a baseline zBMI*

The estimated parameters $\hat{\beta}_{0}$ and $\hat{\boldsymbol{\beta}}$ in equation (1) can be used to predict zBMI for a given composition. For example, to predict a baseline/reference zBMI using the mean daily movement behaviour composition, the compositional values $\left( \bar{\mathrm{Sleep}},\bar{\mathrm{ST}},\bar{\mathrm{LPA}},\bar{\mathrm{MVPA}} \right)$ in minutes (where $\bar{x}$ is the mean value of $x$, and ST = sedentary time, LPA = light-intensity physical activity, MVPA = moderate-to-vigorous-intensity physical activity) are used in the following equation:

$$\hat{\bar{y}}= \hat{\beta}_{0}+{\hat{\boldsymbol{\beta}}}^{T}ilr\left( \bar{\mathrm{Sleep}},\bar{\mathrm{ST}},\bar{\mathrm{LPA}},\bar{\mathrm{MVPA}} \right) (2)$$

*Predicting zBMI for a reallocated new composition*

We predict zBMI for a new composition where time has been reallocated from one behaviour to another, while remaining behaviours are kept constant. For example, a new composition, where 15 min has been reallocated from ST to sleep would be:

$\left( \bar{\mathrm{Sleep}}+15, \bar{\mathrm{ST}}-15,\bar{\mathrm{LPA}},\bar{\mathrm{MVPA}} \right)$.

This new composition is used in the predictive model as follows:

$${\hat{\bar{y}}}_{\left( +15,-15,0,0 \right)}= \hat{\beta}_{0}+{\hat{\boldsymbol{\beta}}}^{T}ilr\left( \bar{\mathrm{Sleep}_{+15}},\bar{\mathrm{ST}_{-15}}, \bar{\mathrm{LPA}},\bar{\mathrm{MVPA}} \right) (3)$$

*Predicting the expected difference in zBMI: comparing a reallocated composition to the mean composition*

The calculation of the difference in predicted zBMI for the movement behaviour compositions in Equation (3) and Equation (2) simply requires the following calculation,

$${\hat{\bar{y}}}_{\left( +15,-15,0,0 \right)}- \hat{\bar{y}} (4)$$

This approach can be used to predict zBMI for any reallocation between movement behaviours from baseline.

Example R code to produce a value for Equation (4) is presented below (requires the R package *Compositions*).

*# load the Compositions package*

**library**(Compositions)

*# create the composition*

comp <- **cbind**(sleep,ST,LPA,MVPA) *#variable are in min/day*

*# treat it as compositional variable*

comp <- **acomp**(comp)

*# create the ilr multiple linear regression model, using the default ilr transformation contained within the Compositions package*

lm <- **lm**(zBMI ~ **ilr**(comp) + age) *#age is included as an example covariate*

*# calculate the mean composition*

comp.mean <- **mean**(comp)

*# the mean() function is from the Compositions package, therefore mean() calculates the compositional mean (i.e., geometric mean, adjusted to sum to 1).*

*# predict zBMI for the mean composition*

*# age is kept constant at its mean.*

mean.pred <- **predict**(lm, newdata=**list**(comp=comp.mean, age=**mean**(age)))

*# now create a new composition, where 15 min of sedentary time are reallocated to sleep.*

*# because the mean composition was adjusted to 1 when it was designated as acomp(), i.e., it is expressed as proportions,*

*# the amount of time reallocated (15 min) must also be expressed as a proportion.*

*# The reallocated time therefore must be expressed as 15/1440.*

new.comp <- **acomp**(comp.mean **+c**(15/1440, -15/1440, 0, 0))

*# predict zBMI for the new composition*

pred <- **predict**(lm, newdata=**list**(comp=new.comp, age=**mean**(age)))

*# the estimated difference in zBMI associated with the above time reallocation is:*

pred - mean.pred
