# Supplementary material for: Fitness, fatness and the reallocation of time between children’s daily movement behaviours: an analysis of compositional data
Source: Int J Behav Nutr Phys Act. 2017 May 10;14:64. doi: 10.1186/s12966-017-0521-z (PMC5424384; doi:10.1186/s12966-017-0521-z)
Supplement: Supplementary file 2 — Variation matrices of weight-status subgroups. (DOCX 13 kb) [file 12966_2017_521_MOESM2_ESM.docx]

**Additional file 2. Variation Matrices of Weight-Status Subgroups**

Compositional variation matrix of time spent by the underweight sample in sleep, ST, LPA, and MVPA

|  | Sleep | ST | LPA | MVPA |
| --- | --- | --- | --- | --- |
| Sleep | 0 | 0.024 | 0.063 | 0.231 |
| ST | 0.024 | 0 | 0.061 | 0.299 |
| LPA | 0.063 | 0.061 | 0 | 0.177 |
| MVPA | 0.231 | 0.299 | 0.177 | 0 |

Note. ST, Sedentary Time; LPA, Light Physical Activity; MVPA, Moderate-to-Vigorous Physical Activity.

Compositional variation matrix of time spent by the normal-weight sample in sleep, ST, LPA, and MVPA

|  | Sleep | ST | LPA | MVPA |
| --- | --- | --- | --- | --- |
| Sleep | 0 | 0.030 | 0.036 | 0.191 |
| ST | 0.030 | 0 | 0.079 | 0.378 |
| LPA | 0.036 | 0.079 | 0 | 0.200 |
| MVPA | 0.191 | 0.378 | 0.200 | 0 |

Note. ST, Sedentary Time; LPA, Light Physical Activity; MVPA, Moderate-to-Vigorous Physical Activity.

Compositional variation matrix of time spent by the overweight/obese sample in sleep, ST, LPA, and MVPA

|  | Sleep | ST | LPA | MVPA |
| --- | --- | --- | --- | --- |
| Sleep | 0 | 0.028 | 0.027 | 0.336 |
| ST | 0.028 | 0 | 0.106 | 0.446 |
| LPA | 0.027 | 0.106 | 0 | 0.310 |
| MVPA | 0.336 | 0.446 | 0.310 | 0 |

Note. ST, Sedentary Time; LPA, Light Physical Activity; MVPA, Moderate-to-Vigorous Physical Activity.
