# Supplementary material for: Fitness, fatness and the reallocation of time between children’s daily movement behaviours: an analysis of compositional data
Source: Int J Behav Nutr Phys Act. 2017 May 10;14:64. doi: 10.1186/s12966-017-0521-z (PMC5424384; doi:10.1186/s12966-017-0521-z)
Supplement: Supplementary file 3 — Predicted means for fatness and fitness used as the starting point for isotemporal substitutions. (DOCX 12 kb) [file 12966_2017_521_MOESM3_ESM.docx]

**Additional file 3**

**Predicted means for fatness and fitness used as the starting point for isotemporal substitutions**.

|  | All children  n=169 | Underweight  n=15 | Normal weight  n=115 | Overweight/obese  n=39 |
| --- | --- | --- | --- | --- |
| zBMI | 0.56 | -1.87 | 0.14 | 2.06 |
| %WHtR | 46.1 | 41.6 | 42.8 | 51.0 |
| VO_2_ peak* | 47.5 | 49.6 | 48.6 | 41.6 |

Note. zBMI, Body Mass Index z-score; CRF, cardiorespiratory fitness; VO_2_ peak, peak oxygen uptake.

Predicted values are for weight-status subgroup mean daily activity composition, and mean age and median sex, IMD decile (*and mean zBMI for VO_2_ peak estimate)
