# Supplementary material for: Fitness, fatness and the reallocation of time between children’s daily movement behaviours: an analysis of compositional data
Source: Int J Behav Nutr Phys Act. 2017 May 10;14:64. doi: 10.1186/s12966-017-0521-z (PMC5424384; doi:10.1186/s12966-017-0521-z)
Supplement: Supplementary file 4 — Differences in predicted zBMI associated with time reallocations for the full sample. (DOCX 15 kb) [file 12966_2017_521_MOESM4_ESM.docx]

**Additional file 4. Differences in predicted zBMI associated with time reallocations for the full sample**

| Minutes reallocated | 2.5 | 5 | 7.5 | 10 | 12.5 | 15 | 17.5 | 20 | 22.5 | 25 |
| --- | --- | --- | --- | --- | --- | --- | --- | --- | --- | --- |
| Increase ST, decrease sleep | -0.01 | -0.02 | -0.03 | -0.03 | -0.04 | -0.05 | -0.06 | -0.07 | -0.08 | -0.09 |
| Increase sleep, decrease ST | 0.01 | 0.02 | 0.03 | 0.03 | 0.04 | 0.05 | 0.06 | 0.07 | 0.08 | 0.09 |
| Increase MVPA, decrease LPA | -0.10 | -0.19 | -0.27 | -0.35 | -0.42 | -0.49 | -0.56 | -0.62 | -0.68 | -0.74 |
| Increase LPA, decrease MVPA | 0.11 | 0.22 | 0.36 | 0.51 | 0.68 | 0.89 | 1.14 | 1.48 | 1.99 | 3.05 |
| Increase LPA, decrease sleep | 0.00 | 0.00 | 0.00 | 0.01 | 0.01 | 0.01 | 0.01 | 0.01 | 0.01 | 0.01 |
| Increase sleep, decrease LPA | 0.00 | 0.00 | -0.01 | -0.01 | -0.01 | -0.01 | -0.01 | -0.01 | -0.02 | -0.02 |
| Increase MVPA, decrease ST | -0.09 | -0.17 | -0.24 | -0.31 | -0.37 | -0.43 | -0.49 | -0.54 | -0.59 | -0.63 |
| Increase ST, decrease MVPA | 0.10 | 0.20 | 0.33 | 0.47 | 0.63 | 0.83 | 1.07 | 1.40 | 1.91 | 2.95 |
| Increase MVPA, decrease sleep | -0.10 | -0.18 | -0.27 | -0.34 | -0.41 | -0.48 | -0.55 | -0.61 | -0.66 | -0.72 |
| Increase sleep, decrease MVPA | 0.11 | 0.22 | 0.35 | 0.50 | 0.67 | 0.88 | 1.13 | 1.47 | 1.98 | 3.04 |
| Increase LPA, decrease ST | 0.01 | 0.02 | 0.03 | 0.04 | 0.05 | 0.06 | 0.07 | 0.08 | 0.09 | 0.10 |
| Increase ST, decrease LPA | -0.01 | -0.02 | -0.03 | -0.04 | -0.05 | -0.06 | -0.07 | -0.08 | -0.09 | -0.10 |

Note. zBMI, Body Mass Index z-score; ST, Sedentary Time; LPA, Light Physical Activity; MVPA, Moderate-to-Vigorous Physical Activity

Estimation of change in predicted zBMI when the behavior in the rows substitutes the behavior in the columns, at the mean daily activity composition.

Analysis adjusted for IMD decile, age, and sex.
