# Supplementary material for: Fitness, fatness and the reallocation of time between children’s daily movement behaviours: an analysis of compositional data
Source: Int J Behav Nutr Phys Act. 2017 May 10;14:64. doi: 10.1186/s12966-017-0521-z (PMC5424384; doi:10.1186/s12966-017-0521-z)
Supplement: Supplementary file 5 — Differences in predicted %WHtR associated with time reallocations for the full sample. (DOCX 14 kb) [file 12966_2017_521_MOESM5_ESM.docx]

**Additional file 5. Differences in predicted %WHtR associated with time reallocations for the full sample**

| Minutes reallocated | 2.5 | 5 | 7.5 | 10 | 12.5 | 15 | 17.5 | 20 | 22.5 | 25 |
| --- | --- | --- | --- | --- | --- | --- | --- | --- | --- | --- |
| Increase ST, decrease sleep | 0.0 | 0.0 | 0.0 | -0.1 | -0.1 | -0.1 | -0.1 | -0.1 | -0.1 | -0.2 |
| Increase sleep, decrease ST | 0.0 | 0.0 | 0.0 | 0.1 | 0.1 | 0.1 | 0.1 | 0.1 | 0.1 | 0.2 |
| Increase MVPA, decrease LPA | -0.6 | -1.2 | -1.7 | -2.2 | -2.7 | -3.1 | -3.5 | -3.9 | -4.3 | -4.7 |
| Increase LPA, decrease MVPA | 0.7 | 1.4 | 2.2 | 3.1 | 4.2 | 5.5 | 7.0 | 9.1 | 12.2 | 18.6 |
| Increase LPA, decrease sleep | 0.1 | 0.1 | 0.2 | 0.2 | 0.3 | 0.3 | 0.4 | 0.5 | 0.5 | 0.6 |
| Increase sleep, decrease LPA | -0.1 | -0.1 | -0.2 | -0.2 | -0.3 | -0.4 | -0.4 | -0.5 | -0.5 | -0.6 |
| Increase MVPA, decrease ST | -0.5 | -1.0 | -1.5 | -1.9 | -2.3 | -2.7 | -3.0 | -3.3 | -3.6 | -3.9 |
| Increase ST, decrease MVPA | 0.6 | 1.2 | 2.0 | 2.8 | 3.8 | 5.0 | 6.5 | 8.5 | 11.5 | 17.9 |
| Increase MVPA, decrease sleep | -0.6 | -1.1 | -1.5 | -2.0 | -2.4 | -2.7 | -3.1 | -3.4 | -3.8 | -4.1 |
| Increase sleep, decrease MVPA | 0.6 | 1.3 | 2.0 | 2.9 | 3.9 | 5.1 | 6.6 | 8.7 | 11.7 | 18.0 |
| Increase LPA, decrease ST | 0.1 | 0.1 | 0.2 | 0.3 | 0.4 | 0.4 | 0.5 | 0.6 | 0.7 | 0.7 |
| Increase ST, decrease LPA | -0.1 | -0.2 | -0.2 | -0.3 | -0.4 | -0.5 | -0.5 | -0.6 | -0.7 | -0.8 |

Note. ST, Sedentary Time; LPA, Light Physical Activity; MVPA, Moderate-to-Vigorous Physical Activity

Estimation of change in predicted %waist:height when the behavior in the rows substitutes the behavior in the columns, at the mean daily activity composition. Analysis adjusted for IMD decile, age, and sex.
