# Supplementary material for: Fitness, fatness and the reallocation of time between children’s daily movement behaviours: an analysis of compositional data
Source: Int J Behav Nutr Phys Act. 2017 May 10;14:64. doi: 10.1186/s12966-017-0521-z (PMC5424384; doi:10.1186/s12966-017-0521-z)
Supplement: Supplementary file 8 — STROBE checklist. (DOCX 30 kb) [file 12966_2017_521_MOESM8_ESM.docx]

STROBE Statement—Checklist of items that should be included in reports of ***cross-sectional studies***

|  | Item No | Recommendation |
| --- | --- | --- |
| **Title and abstract** | 1 | (*a*) THE CROSS-SECTIONAL DESIGN IS REFERRED TO IN THE ABSTRACT |
|  |  | (*b*) ALL REQUIRED INFORMATION IS PROVIDED IN THE ABSTRACT |
| Introduction | | |
| Background/rationale | 2 | THIS INFORMATION IS PROVIDED ON P3-4 |
| Objectives | 3 | STUDY AIM STATED ON P5 |
| Methods | | |
| Study design | 4 | REFERENCE IS MADE TO THE CROSS-SECTIONAL DESIGN ON P5 |
| Setting | 5 | THIS INFORMATION IS PROVIDED ON P5 |
| Participants | 6 | THIS INFORMATION IS PROVIDED ON P5 |
| Variables | 7 | THIS INFORMATION IS PROVIDED ON P5-7 |
| Data sources/ measurement | 8* | THIS INFORMATION IS PROVIDED ON P7-8 |
| Bias | 9 | ALL MEASURES WERE OBJECTIVE WHICH REMOVED SOCIAL DESIRABILITY BIAS ON BEHALF OF THE PARTICIPANTS. THE PARTICIPATION RATE WAS 75% WHICH SUGGESTS THAT SELECTION BIAS WAS MINIMAL. FURTHER, THERE WERE NO DIFFERENCES BETWEEN PARTICIPANTS INCLUDED/EXCLUDED FROM THE ANALYTICAL SAMPLE WHICH SUGGESTS BIAS WAS MINIMISED IN RELATION TO COMPLIANCE TO ACTIVITY MONITORING PROTOCOL |
| Study size | 10 | SAMPLE SIZE WAS DETERMINED BY THE NUMBER OF PARTICIPATING SCHOOLS AND CONSENTING PARTICIPANTS |
| Quantitative variables | 11 | QUANTITATIVE ANALYSIS IS DESCRIBED ON P8-10 |
| Statistical methods | 12 | (*a*) QUANTITATIVE ANALYSIS IS DESCRIBED ON P8-10 |
|  |  | (*b*) QUANTITATIVE ANALYSIS IS DESCRIBED ON P8-10 |
|  |  | (*c*) MISSING DATA RESULTING FROM NON-COMPLIANCE TO ACCELEROMETER MONITORING PROTOCOL WERE REMOVED FROM THE ANALYTICAL SAMPLE ON A CASE BY CASE BASIS |
|  |  | (*d*) N/A |
|  |  | (*e*) N/A |
| Results | | |
| Participants | 13* | (a) PARTICIPANT NUMBERS ARE PRESENTED IN TABLE 1 |
|  |  | (b) EXCLUSION FROM THE ANALYTICAL SAMPLE WAS DUE TO ACCELEROMETER WEAR NON-COMPLIANCE |
|  |  | (c) N/A |
| Descriptive data | 14* | (a) PARTICIPANT CHARACTERISTIC ARE PRESENTED IN TABLE 1 |
|  |  | (b) THIS INFORMATION IS CONTAINED IN TABLE 2 |
| Outcome data | 15* | OUTCOME MEASURES ARE DESCRIBED ON P9 |
| Main results | 16 | (*a*) TABLES 2 AND 3 CONTAIN THE GEOMETRIC MEANS AND COMPOSITIONAL VARIATION MATRIX, RESPECTIVELY |
|  |  | (*b*) N/A |
|  |  | (*c*) N/A |
| Other analyses | 17 | N/A |
| Discussion | | |
| Key results | 18 | KEY RESULTS SUMMARISED ON P12-14 |
| Limitations | 19 | LIMITATIONS DISCUSSED ON P19-20 |
| Interpretation | 20 | OVERALL INTERPRETATION OF THE RESULTS IS PRESENTED IN THE CONCLUSIONS ON P20 |
| Generalisability | 21 | GENERALISABILITY IS REFERRED TO ON P20 |
| Other information | | |
| Funding | 22 | FUNDING INFORMATION IS PRESENTED IN THE DECLARATIONS SECTION ON P21 |

*Give information separately for exposed and unexposed groups.

**Note:** An Explanation and Elaboration article discusses each checklist item and gives methodological background and published examples of transparent reporting. The STROBE checklist is best used in conjunction with this article (freely available on the Web sites of PLoS Medicine at http://www.plosmedicine.org/, Annals of Internal Medicine at http://www.annals.org/, and Epidemiology at http://www.epidem.com/). Information on the STROBE Initiative is available at www.strobe-statement.org.
